# Recent Development in X-Ray Imaging Technology: Future and Challenges

**DOI:** 10.34133/2021/9892152

**Published:** 2021-12-26

**Authors:** Xiangyu Ou, Xue Chen, Xianning Xu, Lili Xie, Xiaofeng Chen, Zhongzhu Hong, Hua Bai, Xiaowang Liu, Qiushui Chen, Lin Li, Huanghao Yang

**Affiliations:** ^1^MOE Key Laboratory for Analytical Science of Food Safety and Biology, College of Chemistry, Fuzhou University, Fuzhou 350108, China; ^2^Frontiers Science Center for Flexible Electronics, Xi'an Institute of Flexible Electronics (IFE) and Xi'an Institute of Biomedical Materials & Engineering, Northwestern Polytechnical University, 127 West Youyi Road, Xi'an 710072, China; ^3^Fujian Science & Technology Innovation Laboratory for Optoelectronic Information of China, Fuzhou 350108, China

## Abstract

X-ray imaging is a low-cost, powerful technology that has been extensively used in medical diagnosis and industrial nondestructive inspection. The ability of X-rays to penetrate through the body presents great advances for noninvasive imaging of its internal structure. In particular, the technological importance of X-ray imaging has led to the rapid development of high-performance X-ray detectors and the associated imaging applications. Here, we present an overview of the recent development of X-ray imaging-related technologies since the discovery of X-rays in the 1890s and discuss the fundamental mechanism of diverse X-ray imaging instruments, as well as their advantages and disadvantages on X-ray imaging performance. We also highlight various applications of advanced X-ray imaging in a diversity of fields. We further discuss future research directions and challenges in developing advanced next-generation materials that are crucial to the fabrication of flexible, low-dose, high-resolution X-ray imaging detectors.

## 1. Introduction

X-rays are a type of ionizing radiation with a wavelength ranging from 0.01 to 10 nm [1, 2]. When X-rays travel through a matter, they are transmitted, absorbed, or scattered. The processes of scattering and absorption depend on the attenuation ability of the matter and are governed by Lambert-Beer's Law (eq. (1)):

(1) $$\begin{array}{c} I=I_{o}e^{-ud}, \end{array}$$

where *I* is the intensity of transmitted X-ray photons, *I*_o_ is the initial intensity of X-ray photons, *μ* is the linear attenuation coefficient, and *d* is the thickness of the matter [3–6]. The attenuation ability is dominated by a combination of the photoelectric effect, Compton scattering, and Rayleigh scattering [7]. Their ratios are determined by both the nature of the matter and the energy of incident X-rays. Typically, in a low-energy X-ray region, X-ray photons are mainly absorbed by the object through the photoelectric effect, while the Compton scattering is dominant in low-*Z* materials and high-energy photons [8, 9].

The excellent penetration ability of X-rays has made X-ray imaging a powerful medical imaging modality [10]. The advances in X-ray imaging have stimulated the progress in diagnostic radiography technologies, physically describing the skeleton, including fractures, luxation, bone disease, and the location of foreign matters [11, 12]. Such imaging information is particularly useful for guiding the surgery [13]. Apart from the medical applications, X-ray imaging is further extensively used for nondestructive industrial and safety inspection [14]. Undoubtedly, the development of X-ray imaging for over a century has promoted the advancement of a wide range of disciplines from fundamental researches to practical applications.

**Figure 1:**
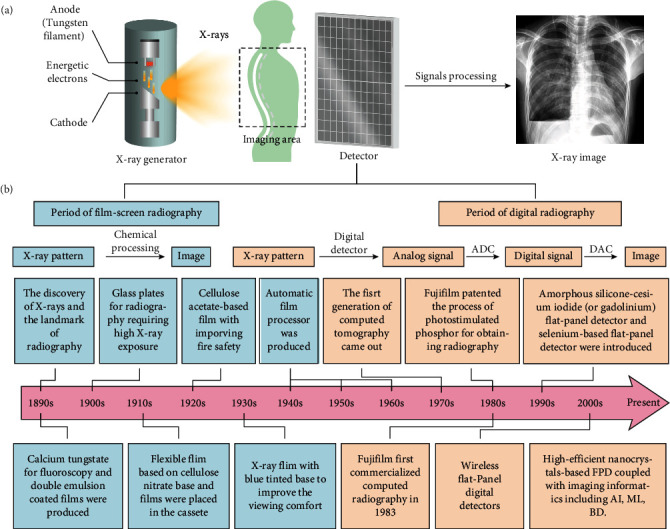
(a) Schematic illustration of an X-ray imaging system. The system constitutes an X-ray generator and an X-ray detector with a signal processing system. X-ray beam produced by the X-ray generator passes through the object (e.g., patient's chest) to arrive at the X-ray detector, followed by the signal processing to produce a visible image. (b) The development of X-ray radiography with the evolution of X-ray detectors. The development can be mainly divided into film-screen radiography and digital radiography. The film-screen radiography converts a latent X-ray pattern into a visible image through tedious chemical processing, whereas digital radiography goes through a series of signal conversions to obtain the X-ray image. ADC: analog-to-digital conversion; DAC: digital-to-analog conversion; AI: artificial intelligence; ML: machine learning; DB: big data.

An X-ray imaging system typically comprises an X-ray generator and an X-ray imaging detector (Figure 1(a), left panel) [15]. The X-ray generator is made of two electrodes sealed into an evacuated chamber. Once powered on, the cathode made of tungsten filament can produce energic electrons through a thermionic effect when it is heated to 2200°C by the electric current. When an accelerating voltage is applied, X-rays are produced during energy changes of fast-moving electrons when they collide and interact with the anode material under a vacuum. The lost energies are converted into bremsstrahlung and characteristic X-rays. Typically, 80% of the X-ray photons emitted by the diagnostic X-ray generator are bremsstrahlung [16, 17]. The output X-ray spectrum is affected by accelerating voltage, filament heating voltage and current, and cathode materials.

The X-ray imaging system converts the X-ray photons transmitted from the object into a visible image that can be used for evaluating the internal structures. The X-ray detector is placed behind objects to record the transmitted X-rays for producing an X-ray pattern (Figure 1(a), right panel). This pattern is subsequently converted into a visible two-dimensional (2D) radiographic image or three-dimensional radiographic image through tomography. Finally, the contrast-based X-ray images are generated based on the attenuation difference of the objects within the matter towards X-rays [18–20].

In this review, we give a detailed overview of the recent development of X-ray imaging technologies, including film-screen radiography and digital radiography, according to the evolution of X-ray detectors in the imaging system. In each section, we start with a description of the structure of the device and the corresponding working principle. The advantages and disadvantages of each X-ray imaging system are further discussed. This review is ended with a perspective on the further development direction of X-ray radiography.

## 2. Film-Screen Radiography

### 2.1. Substrate Materials

The first X-ray image was taken by a radiographic plate several months after the X-rays discovered by Röntgen, where the finger bones and the ring of his wife were clearly imaged [24]. The radiography manifested its original application in medical diagnosis and was further used for the identification of jewelry and art collection and nondestructive detection of metallic objects in the industry soon. Although photographic plate-based X-ray detectors made of a glass plate coated with a thick layer of light-sensitive emulsion show great promise in radiography, they are fragile, heavy, expensive, and difficult for operation and storage.

The challenges in radiographic plates promoted the development of substitutive substrate materials with flexibility, portability, transparency, and relative thinness. The photographic film consisting of cellulose nitrate and emulsion was first developed to replace the glass plate. Since the cellulose nitrate was flammable, nonflammable cellulose triacetate materials such as polyester materials were used for X-ray film instead [25].

### 2.2. X-Ray Film and Cassette

**Figure 2:**
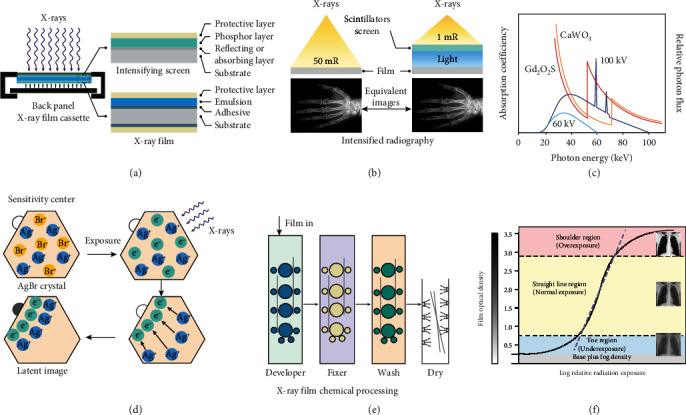
Film-screen radiography system. (a) Scheme of an X-ray film cassette and profiles of intensifying screen and X-ray film. The lightweight film cassette made of metal materials features a carbon fiber protective shield, intensifying screens, X-ray film, and a back panel. (b) The comparison of X-ray film direct exposure to X-rays (left) with that of X-ray film in combination with a scintillators screen. (c) X-ray absorption spectra of gadolinium oxysulphide (GOS, red), calcium tungsten (CaWO_4_, orange), and X-ray emission spectra of 60 kV (sky blue) and 100 kV (dark blue) X-rays. The atomic number of rare-earth elements ranges from 57 to 70 with the *K*-edge between 39 and 61 keV. (d) Upon X-ray irradiation, the free silver ions aggregate in negatively charged sensitivity centers to acquire an electron, forming the latent image region. (e) The latent image in the film was converted into the visible image through photochemical processing including development, fixation, washing, and drying. (f) The characteristic curve of the film-screen system in response to X-ray exposure, which can be divided into three parts including the toe region (blue), the straight-line region (yellow), and the shoulder region (red). Notably, base plus fog is the background intensity of the unexposed film produced by accident light irradiation. The optical intensity is a function of X-ray exposure plotted on a logarithmic scale. (a) is reprinted with permission from ref. [21], copyright 2013 *Author*. (c) is reprinted with permission from ref. [22], copyright 2008 *Elsevier Ltd*. (f) is reprinted with permission from ref. [23], copyright 2019 *Elsevier Springer Nature Singapore Pte Ltd*.

As illustrated in Figure 2(a), the X-ray cassette has a flat, lightproof metal box consisting of an intensifying screen and a radiographic film. The top protective layer made of opaque carbon fiber shows nearly no radiation absorption. The back layer of the cassette utilizing a thin layer of lead with an atomic number of 82 is designed to avoid potential backscattered radiation from the transmitted X-rays [21]. The X-ray film consists of the protective layer, emulsion, adhesive, and polymer substrate. The substrate is coated with a thick layer of photosensitive emulsion on both sides to increase the X-ray absorption for mitigating blurring. Typically, the emulsion layer is made of innumerable silver-halide compounds mixed with gelatin material [26, 27]. However, the sensitivity of X-ray imaging is very limited when the emulsion is directly exposed to X-rays, and this is largely attributed to its low X-ray absorption efficiency.

### 2.3. Intensifying Screen and Its Composition

Although silver halide crystals can be directly exposed by the X-rays, a high dosage of X-rays with a risk of irradiation damage is required for qualified X-ray imaging. To reduce the radiation dose, a fluorescent intensifying screen made of scintillators was introduced for converting X-rays into ultraviolet to visible (UV-Vis) light to sensitize the radiographic film [28]. Figure 2(b) indicates that a radiographic film coupling with an intensifying screen substantially decreases the X-ray exposure as compared to exposing the radiographic film directly. Of which, 95% of the silver halide crystals are efficiently reduced via the visible light produced by intensifying screen, and the remaining are reduced by the direct interaction with X-rays.

The scintillators act as the energy mediator for intensifying screens, and thus their performance plays a significant role in determining image quality. Over the decades, high-quality scintillators are developed to reduce X-ray exposure. Calcium tungstate (CaWO_4_), a class of scintillator emitting blue light under X-ray exposure, is utilized for X-ray energy conversion by Thomas Edison thanks to its strong X-ray stopping power and high X-ray scintillation efficiency. However, the absorption coefficiency of CaWO_4_ is not optically matched to the spectra at 60-100 keV X-rays, as demonstrated in Figure 2(c) [22]. The development of scintillators with improved absorption in low X-ray energy is desired. Rare-earth-activated materials with high atomic numbers in the range from 57 to 70 (*K*-edges between 39 and 61 keV) exhibit high X-ray attenuation and scintillation efficiency, such as lanthanum bromide oxide, lanthanum oxysulfide, and GOS. A rare-earth-based scintillating screen has a low radiation dosage of about 2-3 times less than the CaWO_4_ screen with superior X-ray image quality.

### 2.4. Chemical Processing for the Radiographic Film

The chemical process to capture an X-ray image using a radiographic film involves the formation of a latent image and then developing an X-ray image [29, 30]. Silver-halide crystals have a cubic phase structure with lattice points occupied by negatively charged bromide (or iodide) ions and positively charged silver ions. The silver halides absorb the photon energy of visible light or X-rays and release electrons to form electron-hole pairs, and the released electrons combine with silver ions in the photosensitive center composed of defects (point defects, dislocations, etc.) in the crystals to produce neutral silver atoms. As a result, silver atoms accumulate to form photosensitive spots, thereby forming a latent image (Figure 2(d)).

After the X-ray film exposure, it was chemically processed to obtain a visible image that can be displayed by transillumination on an appropriate view box for further evaluation. As shown in Figure 2(e), this processing involves development, fixation, washing, and drying. [31]. During a typical development process, electrons from the developer migrate to sensitized grains and convert the silver ions into black silver particles to form a visible image on the film. After leaving the developer solution, the unexposed silver bromide on the film is dissolved and removed in the fixer solution containing acetic acid and sodium thiosulfate. At the same time, sodium sulfite and aluminum chloride in the fixer solution are used as a preservative and a hardener, respectively. Finally, the processed film is washed to remove the fixer solution through a water bath and dried in a chamber in which the hot air is circulating [32].

### 2.5. Characteristic Curve of a Radiographic Film

The performance of an X-ray film is strongly related to the radiation exposure on a logarithm scale. Contrast is the difference in luminance or color, making an object distinguishable. For a specific radiographic film, the contrast depends on the design of the film, the amount of exposure, and the chemical processing conditions. As described in Figure 2(f), there are three different regions in the characterization curve including the toe region (blue), straight-line region (yellow), and shoulder region (red) from the bottom to the top. The toe and shoulder regions with shallow slopes correspond to underexposure and overexposure, respectively. The overexposed image in the shoulder region implies that the silver ions have been reduced to silver atoms, whereas the image will be underexposed and generally useless in the toe region. The normal exposure region is the nearly straight-line portion where a well-exposed image is produced (with a density between 0.5 and 2.75) [23].

## 3. Computed Radiography

### 3.1. Substitution of Film-Screen Radiography by Computed Radiography

**Figure 3:**
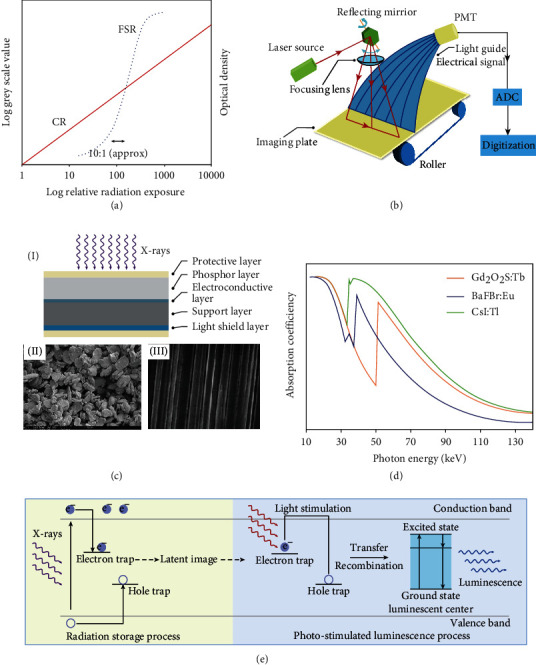
Computed radiography and its imaging mechanism. (a) The characteristic exposure curve of film-screen radiography (FSR, blue dotted line) and computed radiography (CR, red line). The film-screen radiography shows a linear exposure range of 10 : 1, and the digital radiography shows a linear exposure of 10^4^ : 1. (b) Schematic diagram showing a typical computed radiography reader system and the corresponding image readout process. (c) Schematic diagram of the cross-section of the imaging plate (I). Scanning electron microscope (SEM) image of the structured (III) and unstructured (II) phosphors. (d) X-ray absorption spectra of thallium-doped cesium iodide (CsI: Tl, green), terbium-doped gadolinium oxysulphide (GOS: Tb, orange), and europium-doped barium fluobromide (BaFBr: Eu^2+^, blue) as a function of X-ray photon energy. (e) The physical process of photostimulation using BaFBr: Eu^2+^ phosphors. It can be divided into two steps, including radiation storage (light yellow) and photostimulated luminescence (light blue). The X-rays penetrating the object are absorbed by phosphors, creating a lot of electron-hole pairs, which subsequently migrate to emitting centers or are captured by metastable energy traps. Electrons and holes in the metastable energy traps absorb low-energy laser irradiation to overcome the energy barrier, escaping from the traps, followed by recombination at emitting centers to generate photostimulated luminescence. (a, b) are reprinted with permission from ref. [38], copyright 2007 *Elsevier Ltd*. (d) is reprinted with permission from ref. [41], copyright 2007 *American College of Radiology*.

Although conventional film-screen radiography has contributed extraordinarily to medical diagnosis and industrial inspection since 1895, it suffered from several limitations, including complicated chemical processing, low automatic processing efficiency, high costs of film materials, time and labor consumption, inconvenient images storage and communications, and environmental pollution [33–35]. To this end, digital radiography was developed to replace film-screen radiography. This new technology involves using a digital detector to convert X-ray patterns into digital signals which are subsequently processed and displayed on the screen for observation. It mainly comprises imaging acquisition, laser stimulation, electric signal processing, image display, postprocessing, storage, and communication components [36]. In addition, when compared with film-screen radiography (FSR, blue dotted line), computed radiography shows an improved linear exposure range (10^4^ : 1), suggesting a wide range of radiation exposure (Figure 3(a)) [37, 38].

### 3.2. Image Readout Process of Computed Radiography

Computed radiography, firstly introduced by Fujifilm in 1983, is a technology on the basis of recording the latent image in a photostimulable phosphor-contained imaging plate through laser-light stimulation [39, 40]. A computed radiography system mainly comprises two components, including an imaging plate and a computed radiography reader. They are designed to store the latent image of the X-ray attenuation pattern in the imaging plate and to read out the stored latent image through the reader, respectively. On a separate note, the computed radiography reader (point-scan, laser flying spot) consists of a set of subcomponents, such as the stimulating laser source, reflecting mirror, light collection guide, and photomultiplier tubes (PMT) [38].

During a computed radiographic imaging process, an X-ray attenuation pattern transmitted from the object is stored in photostimulable phosphors embedded into the imaging plate, leaving a latent image [42]. Then, a laser raster scanning can be used to read out the stored imaging information through releasing the photostimulated luminescence using photomultiplier tubes. Thereafter, in situ generated luminescence signals were converted to electric signals for generating high-quality images by an analog-to-digital converter (Figure 3(b)). The imaging plate can be repeatedly used by removing the residual energy within the phosphors through intense laser light [43]. However, the residual energy in the imaging plate cannot be completely erased since it is hard to release all the trapped energy in phosphors by a laser scanning. It is essential to extend the erasure time and increase the erasure cycle to eliminate all the residual energy for further use.

### 3.3. The Composition of the Imaging Plate and the Property of Phosphors

In computed radiography, an imaging plate is used to replace the intensifying screen and photographic film. As shown in Figure 3(c), the protective layer on both sides prevents the imaging plate from being scratched, ensuring the durability of the imaging plate and allowing laser transmission. The phosphors layer, which can store the latent image, is made of phosphors mixed with a polymer binder. The electroconductive layer prevents the image quality from degrading by static electricity. The support layer in the middle endowed the imaging plate with a certain mechanical strength. The backscatter radiation is blocked by the light shield layer with a lead backing.

Regarding the phosphors within the imaging plate, there are three prerequisites: first, the emission of the phosphors is required to overlap with the maximum quantum efficiency wavelength of the photomultiplier; second, the irradiated phosphors should exhibit a fast response to the laser scanning for fast imaging; third, no significant signal deterioration for at least 8 h is required for practical use. However, there are almost no phosphors that can simultaneously satisfy the above three aspects at the same time. Among them, BaFX: Eu^2+^ (*X* = Cl, Br, or I) phosphor family has been extensively studied. Although RbBr: Tl^+^ and CsBr: Eu^2+^ can also be used as phosphors in imaging plates benefitting from their easy preparation in the form of a needle-like structure array, the quick latent image loses (tens of seconds) limit their further use for computed radiography systems (Figure 3(c), (I)).

The commercial materials for the phosphor layer are BaFBr: Eu^2+^ and CsBr: Eu^2+^ (Figure 3(c), (II)). Trace amounts of Eu^2+^ activators are doped to replace Ba^2+^ ions in the crystal to form the luminescent centers. Such a doping treatment can alter the structure and consequently the physical properties of the photostimulated phosphors [44, 45]. As compared with the rare-earth-based materials used in the screen system, BaFBr: Eu^2+^ shows efficient X-ray absorption in the range from 35 to 50 keV because of low *K*-edge absorption of barium, as presented in Figure 3(d). Beyond this range, either GOS:Tb phosphors or CsI: Tl phosphors display better performance, allowing their widespread application in indirect flat-panel X-ray detectors or optically coupled digital radiography systems [41, 46].

### 3.4. The Mechanism of Photostimulated Luminescence

The possible energy transfer mechanism inside the photostimulated phosphors is illustrated in Figure 3(e). The in situ generated electron-hole pair concentration within phosphors is proportional to the absorbed radiation energy of the host lattice. In addition, X-ray patterns transmitted from the object could interact with halide ions to displace them into interstitial host sites, thus creating halide ion vacancies and interstitials. Electrons and holes are captured by traps, leading to the formation of latent images. Subsequently, the electrons and holes can spontaneously escape from the traps at ambient conditions, resulting in the gradual deterioration of the storing energies. When the scanning laser light is applied, the carriers trapped in the defects absorb enough energy from stimulation light to overcome the energy barrier, moving freely in the crystal until the occurrence of recombination to release their energy to luminescence centers (e.g., Eu^2+^) accompanied by emitting the light-stimulated luminescence. At last, the carriers still trapped should be effectively excited to empty residual energy to prevent the generation of a ghost image in the next use [47, 48].

## 4. Flat-Panel Detector-Based Radiography

### 4.1. The Origin of Flat-Panel-Based Digital Radiography

With the advancement of photolithography and microelectronic fabrication technology, large-area, flat-panel-based digital radiography was developed in the early 1990s [49]. Digital radiography technology converts the incident X-ray photons into electrical charges and reads the images using photoelectric conversion arrays, displaying a faster readout time than computed radiography [50]. Low-dose, real-time X-ray imaging using flat-panel detectors has been widely used for clinical diagnosis, including chest X-rays, dental X-rays, mammography, and lumbar spine X-rays. Digital radiography is also used in industrial inline nondestructive inspection, such as high-resolution analysis of circuit boards for solder joint porosity measurements and defects detection. Moreover, digital radiography has been widely used in X-ray security scanners in train stations and airports for the screening of dangerous goods and prohibited items.

**Figure 4:**
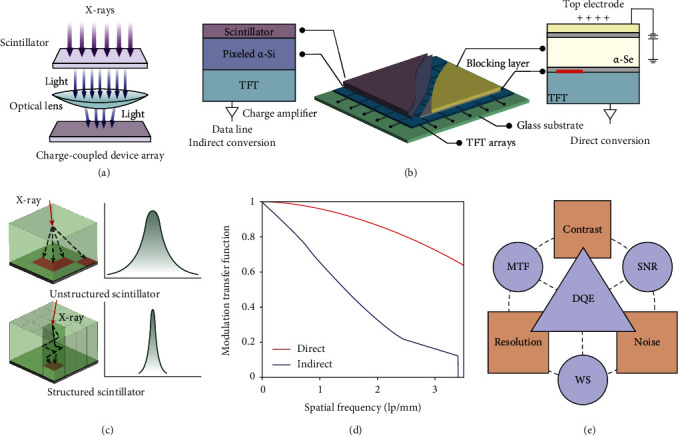
Flat-panel-based digitized radiography and the technical factors influencing imaging quality. (a) Schematic illustration of an optical len-coupled indirect conversion digitized radiography system based on a charge-coupled device. The incident X-rays are converted into UV-Vis light by the scintillators and further into electric signals after being focused by an optical lens and directed to the charge-coupled device array. (b) The schematic illustration of the internal construction of a flat-panel detector (middle panel), which could be classified into indirect conversion flat-panel detector (left panel) and direct conversion flat-panel detector (right panel) based on the X-ray energy conversion modality. For indirect conversion, X-rays transmitting through the scintillator (purple) are converted into UV-Vis light, which is further converted into an electrical charge by the pixelated amorphous silicon photodiodes (*α*-Si; violet), whereas X-ray photons are directly converted into electrical charge in a direct conversion detector. TFT: thin-film transistor. (c) The schematic diagrams (left panel) and line spread function (right panel) of the unstructured and structured scintillators. The X-ray-induced visible luminescence in the unstructured scintillators exhibits a severe scattering in all directions to reduce the imaging spatial resolution, resulting in a wide line spread function. The structured scintillators consist of phosphors in a needle-like structure in favor of reducing the lateral scattering of light, contributing to a narrow line spread function. (d) Comparison of the modulation transfer function (MTF) for direct conversion flat-panel detector (red) and indirect conversion flat-panel detector (blue). (e) Relationships between image quality parameters, including detective quantum efficiency (DQE), modulation transfer function (MTF), signal-to-noise ratio (SNR), and Wiener spectra, and physical image measurements, including contrast, resolution, and noise. Panel (a) is reprinted with permission from ref. [51], copyright 2007 *RSNA*. Panel (c) is reprinted with permission from ref. [56], copyright 2011 *American Institute of Physics*. Panel (e) is reprinted with permission from ref. [57], copyright 2008 *Elsevier Ltd*.

The charge-coupled device-based detector appeared in 1990 was the first large-area flat-panel-based radiography. A charge-coupled device is made of metal-oxide-semiconductor capacitors as a light-sensitive sensor for recording images. In general, a large number of charge-coupled devices are coupled to create a detector array for large-area detection. The incident X-ray photons can be converted into visible luminescence by scintillators (e.g., CsI: Tl, and GOS: Tb). Next, the luminescence is directed to the charge-coupled device array using an optical lens system (Figure 4(a)) [51, 52]. However, the optical lens system can reduce the number of photons reaching the charge-coupled device arrays, which may result in low quantum efficiency and high image noise, and thus lead to poor image quality. Meanwhile, the optical coupling may also cause geometric distortions and light scattering and consequently a reduced imaging spatial resolution. Besides, high-working temperatures give rise to signal noise within the charge-coupled device itself, deteriorating the image quality. Although the electric cooling charge-coupled device could alleviate this effect, it has an unacceptably high cost. In addition, the size limitation of the charge-coupled device and the optical coupling method make it rigid to fabricate a large-area X-ray detector [53].

### 4.2. Evolution of Thin-Film Transistor Array-Based Digital Radiography

By contrast, flat-panel detectors with large-area photoelectric arrays allow the integration with an X-ray energy conversion layer and thin-film transistor (TFT) array-based electronic readout layer [54]. Unlike charge-coupled devices with coupling optical lenses systems, TFT-based flat-panel X-ray detector is capable of achieving low-dose, real-time X-ray imaging through coupling an energy transfer layer and large-area pixelated TFT arrays (Figure 4(b), middle panel)), becoming popular for applications in angiography, radiography, and mammography. According to the difference in the pathway of converting X-ray radiation to charge carriers, flat-panel X-ray detectors are categorized into indirect conversion systems and direct conversion systems [55].

#### 4.2.1. Direct Conversion X-Ray Detector

Direct conversion X-ray flat-panel detector is fabricated by depositing a layer of X-ray-sensitized materials onto pixelated TFT arrays capable of directly converting X-ray photons into electrical charges that allow being transferred to thin-film transistors (Figure 4(b), right panel) [58]. The most commonly used photoconductor material is amorphous selenium (*α*-Se) fabricated by evaporation at high temperatures [59]. Upon X-ray irradiation, the *α*-Se photoconductor can absorb the X-ray energy and convert it into charge carriers which are proportional to the incident X-ray photons. The hole-electron pairs generated in the photoconductor travel along the field lines parallelly with limited lateral diffusion because of the electric field applied in the *α*-Se. Holes can be collected by the positive bias electrode, whereas electrons can be collected by collection electrodes. The charges are stored on the storage capacitor and then are subsequently read out by thin-film transistors. Each pixel is effectively separated by the field-shaping in the *α*-Se layer, contributing to a high-quality X-ray image [60].

#### 4.2.2. Indirect Conversion X-Ray Detector

Indirect conversion flat-panel X-ray detector is made of a layer of scintillator thin-film on the top for X-ray energy conversion, pixelated amorphous silicon (*α*-Si) photodiode arrays adjacent to scintillators, and a TFT array (Figure 4(b), left panel) [61]. When X-ray irradiates the flat-panel X-ray detectors, X-ray photons are converted into visible luminescence by scintillators and subsequently converted into electric charges by the *α*-Si photodiode arrays. Eventually, the electric charges are recorded by a TFT array [62].

The most widely used scintillators are CsI: Tl with a thickness of 150-600 *μ*m and terbium-doped GOS: Tb [63, 64]. The scintillators deposited in indirect flat-panel X-ray detectors can be either unstructured or structured thin-film layers. For the unstructured scintillators, such as GOS: Tb powder crystals (turbid phosphors), the emitted light traveling in the materials may spread to the neighboring pixels, resulting in a reduced spatial resolution. This matter could be overcome by utilizing structure scintillators, like CsI: Tl consisting of discrete and parallel “needles” with 5-10 *μ*m wide [65]. In this case, the X-ray-excited luminescence only travels along with the fiber-like crystal to the photodiodes, which improves the spatial resolution, making unstructured scintillators superior to that achieved by the structured scintillators, as illustrated in Figure 4(c) [56, 66, 67].

### 4.3. Primary Physical Parameters of X-Ray Imaging

A high-quality digital radiographic image is important for accurate testing and diagnosis. The X-ray imaging quality can be evaluated by three primary parameters, including spatial resolution, contrast, and noise (Figure 4(e)). The physical parameters are generally evaluated by measurements of Wiener spectra (WS), modulation transfer function (MTF), and signal-to-noise ratio (SNR) [57].

#### 4.3.1. Spatial Resolution

The ability to distinguish adjacent details in an object and its related sharpness can be defined by spatial resolution. For digital systems, the spatial resolution relates to the pixel size in the matrix, which is crucial to achieving a high spatial resolution for digital X-ray imaging [68]. This parameter could be measured using a narrow slit, a sharp-edged object, and a bar test pattern. In most cases, a line spread function is used for narrow slit imaging. For instance, *α*-Se-based direct conversion flat-panel X-ray detectors exhibit better imaging spatial resolution than that of indirect conversion flat-panel detectors since the former has nearly no light scattering.

#### 4.3.2. Contrast

The contrast is another key parameter used for evaluating X-ray imaging quality. It refers to the relative brightness of two positions in an X-ray image by measuring the characteristic exposure curve of an X-ray imaging system. For producing a useful image, the contrast is described by a dynamic range of an X-ray detector in response to various X-ray dose exposure. When compared with screen-film radiography, digital radiography exhibits a much wider and linear dynamic range, reducing the risk of overexposure or underexposure. Moreover, the differences between specific tissues (e.g., bones and soft tissue) could be reflected in one image through post-processing without further exposure [69].

#### 4.3.3. Noise

The noise signals originating from various sources (e.g. collection element, coupling element, capture element, etc.) are characterized by the variations of signals in an X-ray image of a uniform object [70]. The noise of an X-ray detector is important for determining image quality. The factor of Wiener spectra (WS) is used to measure the noise variation of an X-ray image, indicative of the functional relationship between spatial frequency and the corresponding noise.

#### 4.3.4. Modulation Transfer Function (MTF)

The spatial resolution of an X-ray imaging detector can be measured by the MTF. More specifically, the MTF is used to convert the values of object contrast into contrast intensity levels of an X-ray image [71]. As mentioned above, due to the limited lateral scattering, the MTF for direct conversion flat-panel X-ray detectors is obviously higher than that measured by the typical indirect conversion flat-panel X-ray detectors, as presented in Figure 4(d).

#### 4.3.5. Detective Quantum Efficiency (DQE)

DQE is currently used as the standard measurement to evaluate image quality in radiography and assess the efficiency of an X-ray imaging detector in detecting X-ray photons [72]. Remarkably, the DQE takes into consideration the signal-to-noise ratio (SNR) and the system noise. The DQE indicates the performance of the X-ray imaging detector in terms of X-ray imaging quality and the X-ray radiation dose. The DQE for digital radiography is higher than that for conventional screen-film radiography, indicating that digital radiography can convert a higher proportion of incident radiation into image signals compared to conventional screen-film radiography. In particular, the DQE for CsI: Tl-based indirect flat-panel detector could reach 40-45% at 0.5 lp/mm, while that for computed radiography is generally less than 30% at 0.5 lp/mm.

## 5. Computed Tomography (CT)

### 5.1. The Development of Three-Dimensional (3D) Radiography

**Figure 5:**
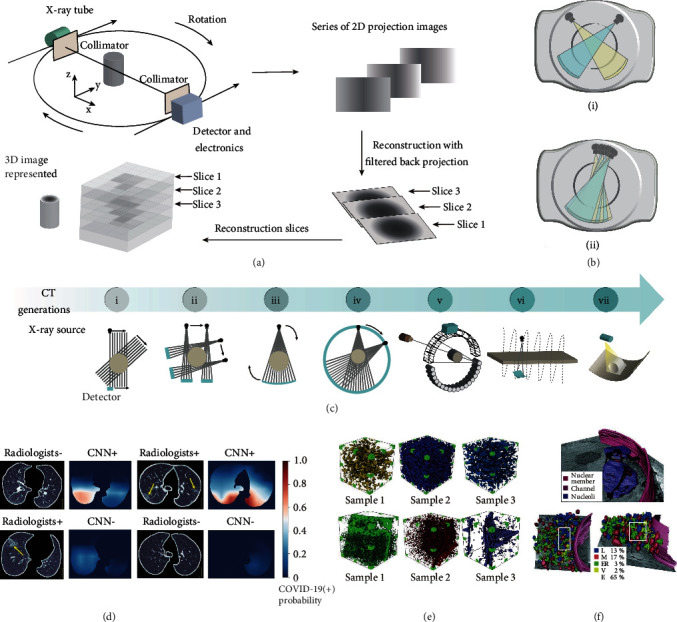
Demonstration of CT. (a) Schematics illustrating the working principle of CT scanning and imaging. The collimated X-ray photons penetrating through the object are recorded using an X-ray detector, which is positioned opposite to the X-ray generator. In a typical CT scanning, the solid-state detectors rotate around the object in synchrony with an X-ray generator to produce a series of 2D projection images. Subsequently, the 2D slice images are obtained after reconstruction with filtered back projection. Eventually, a 3D tomographic image is reconstructed through computer algorithms. (b) The structure of dual-energy CT (i) and multienergy CT (ii). The dual-energy CT or multienergy CT is equipped with two- or multi-X-ray generators, which allow simultaneous acquisition of images under two- or multienergy level X-rays in a single scan. (c) The development history of CT. The evolution of CT has gone through seven generations. (d) Combined diagnosis of CT and an artificial intelligence algorithm deep convolutional neural network (CNN) on four sectioned chest images. CT images are used as input data for the CNN model; subsequently, the output images (right panel) are presented as the heat maps, where red indicates a high risk of COVID-19 infection. (e) The reconstructed 3D porous structure of six coal samples. The pore size distribution, pore volume, porosity, and permeability data could be obtained. (f) The 3D X-ray images showing the volume fraction of organelles (bottom panel) and the nuclear membrane (top panel). L: lysosomes; M: mitochondria; ER: endoplasmic reticulum; V: vesicles; E: external. (d) is reprinted with permission from ref. [76], copyright 2020 *The Author(s), under exclusive license to Springer Nature America, Inc*. (f) is reprinted with permission from ref. [77], copyright 2010 *Nature America, Inc*.

Regarding projection radiography, a large proportion of the depth information is lost since all structural details from a 3D object are projected on a 2D plane X-ray detector, producing an overlapped radiographic image, which will lead to misinterpretation of the internal structures. Fortunately, a new technique named CT was developed to overcome this limitation in the 1970s [73]. As shown in Figure 5(a), series of projection images are acquired from various angles to generate tomographic images. A 3D image is then obtained by reconstructing these tomographic images using computer algorithms [74, 75]. Compared with projection radiography, CT can provide comprehensive 3D anatomical reconstructions and has a greater diagnostic capability.

The first applicable CT scanner, consisting of an X-ray generator and two collimated sodium iodide crystals-photomultiplier detectors, was invented by Godfrey N. Hounsfield in 1968. Hounsfield was awarded the Nobel Prize for his contribution to CT. From then on, seven generations of the CT have developed, including updating the shape of X-ray source from pencil to cone, increasing the number of imaging slices and detectors, and changing the scanning mode from rotation and translation to helical scanning (Figure 5(c)).

The first CT scanner uses a rotate/translate system equipped with an X-ray generator with a pinhole collimator to produce the collimated X-rays (i); the second-generation CT scanner incorporates an X-ray generator, which could produce a narrow, fan-shaped X-ray beam, and increases the X-ray sensor number (ii); the third-generation scanner involves a fan-shaped X-ray beam with an angle ranging between 40 and 60 degrees, which enable scanning the object in a rotated modality (iii); the fourth-generation CT system employs a rotating X-ray tube and a stationary, closed X-ray detector ring to alleviate the ring artifacts produced by the third generation (iv); the fifth-generation CT scanner is composed of no moving parts, and the electron beam is directed around the target ring, allowing for all stationary instrumentation (v); the addition of a slip ring stimulated the development of six-generation CT (vi); the seventh-generation CT scanner consists of a multiple detector array and a cone-shaped X-ray beam (vii).

More importantly, dual- and multienergy CT was allowed to be constructed by equipped dual- and multi-X-ray tubes, permitting to operate at different tube voltages to make dual and multienergy scanning possible (Figure 5(b)). The merits of dual- and multienergy CT lie in the fact that data sets at two different photon spectra can be obtained simultaneously upon a single scanning. Furthermore, the dual-energy algorithm can increase the contrast of bone, which is powerful to directly visualize the iodinated vessels without interference. As a result, dual-energy CT is widely used in angiography to create a virtual noncontrast image.

### 5.2. Application of 3D Radiography

As is presented in Figure 5(d), medical CT scanner is extensively used to screen the size, types, location, and numbers of pulmonary nodules, which could offer an accurate assessment of the risk for further treatment. Recent studies showed that CT is valuable for the COVID-19 diagnosis [76]. CT helps to obtain the pathophysiology characters of COVID-19 infected person, such as consolidations of the lungs and bilateral/peripheral ground-glass opacities. These data provide the most intuitive and precise diagnosis information, thereby greatly enhancing diagnostic efficiency.

Apart from the medical application, CT technology is also introduced to industrial nondestructive inspection in early 1980. Industrial CT is a promising nondestructive tool for characterizing the flaws, inclusions, cracks, and insufficient fusion within the body of materials [78–80]. For instance, the 3D pore structure of coal samples can be obtained through CT to reproduce the precise distribution of coal pore and pore structure (Figure 5(e)). The heterogeneity inside different coal samples can be directly observed, providing insights into the structure-dependent attributes of coal, including gas transport, thermomechanical, and failure behaviors.

As an added benefit, the tomography technology is able to perform 3D imaging of nano- to microsized biological organisms when coupling with X-ray microscopy [81–83]. This combined technology enables the diffraction limit of the conventional microscope to be overcome because of using shorter wavelength X-ray photons. This feature suggests its power in elucidating the detailed structural information of in vivo or ex vivo biological samples with a cellular resolution. Note that an emerging transmission soft X-ray microscope could generate 3D cell imaging at a nanoscale resolution based on the difference in X-ray absorption between organic matter and water, filling the gap between cryoelectron tomography and fluorescence superresolution microscopy (Figure 5(f)) [77].

## 6. X-Ray Microscopy

### 6.1. The Development of X-Ray Microscopy

Optical microscopy is great of significance to study microstructures [84]. Fluorescence microscopy provides an approach to image the structures at a microscale resolution by taking advantage of site-specific fluorescence labeling. However, the imaging resolution of fluorescence microscopy is largely limited by the wavelength of UV-Vis light, as confined by Abbe or Rayleigh laws [85, 86]. The imaging resolution can be significantly enhanced to a few angstroms using an electron beam as the incident light in transmission electron microscopy [87]. This technology shows considerable disadvantages in the observation of biological samples, especially considering the tedious sample preparation process including dehydration, formalin fixation, paraffin-embedding, and section. Besides, the poor penetration depth of electrons in biological samples has a limitation to imaging the sample thickness larger than 100 nm.

**Figure 6:**
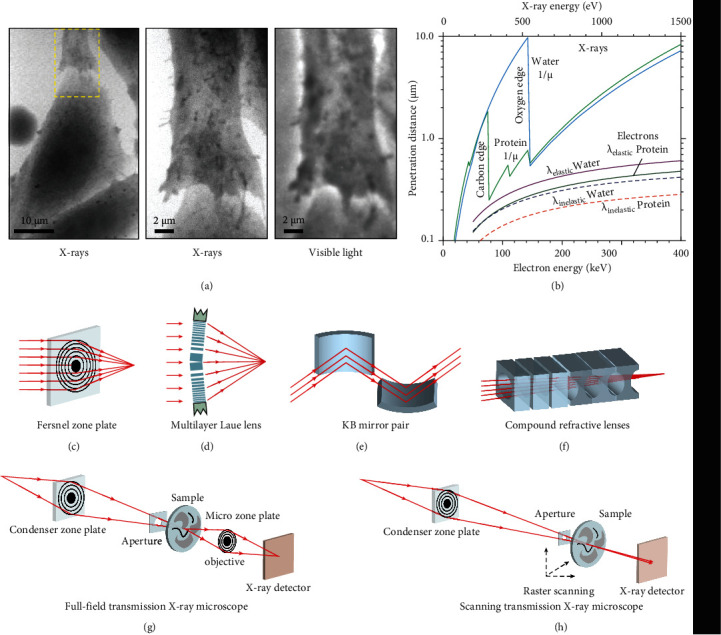
X-ray microscopy setups and their optics. (a) X-ray and optical images of a fibroblast. The area outlined with yellow dashed in the low-magnification image (left panel) is shown at a higher magnification image by the means of X-ray microscopy (middle panel) and optical microscopy (right panel). (b) The penetration distances of X-rays and electrons in water and protein as a function of their energy. The lines from left to right represent attenuation lengths (1/*μ*) of carbon (protein) and oxygen (H_2_O) for X-rays and the mean free paths (*λ*) of H_2_O (elastic scattering), protein (elastic scattering), H_2_O (inelastic scattering), and protein (inelastic scattering), respectively. (c) A Fresnel zone plate is made of several transparent and opaque concentric circulars with radially increasing line density. The central absorbing region is responsible for suppressing the strong zero-order diffraction. (d) One-dimensional multilayer Laue lens for hard X-ray focusing. Alternating layers are fabricated by the thin-film deposition technique to implement thin thickness and ultra-high aspect ratio. (e) A Kirkpatrick-Baez mirror for hard X-ray focusing. Multilayer coatings were designed to increase the angles of operation and to perform photon energy selection. (f) Compound refractive lenses for X-ray focusing at a range of 5-40 keV. A linear array of lenses is manufactured by high-density low-atomic number materials. (g) Full-field transmission X-ray microscopy. A full-field image was projected by a microzone plate onto the X-ray detector. (h) Scanning transmission X-ray microscopy. A zone plate is used to focus coherent X-rays on the sample, whereas an X-ray-sensitive detector is used to capture X-ray images. The sample is mounted on a stage having stepping or piezoelectric driven motors to perform the raster scan. (a, b) are reprinted with permission from ref. [88], copyright 1995 *Cambridge University Press*. (c)–(f) are reprinted with permission from ref. [94], copyright 2010 *Macmillan Publishers Limited*.

By taking advantage of the powerful penetration and nearly no scattering properties of X-rays, the emerging X-ray microscopy techniques break the penetration depth limitation of transmission electron microscopy and allow the intact sample to be imaged without specimen sectioning. The wavelength of X-ray locates at a range of 0.01-10 nm, which is suitable to be used as a light source for imaging biological objects at a very high spatial resolution. It is worth noting that the penetration ability of soft X-rays is much greater than that of electrons. Meanwhile, the water is nearly transparent to X-rays compared to organic compounds at X-ray energy located at 284–540 eV (water window), where the *K* absorption edges of carbon and oxygen are 284 eV and 540 eV, respectively (Figure 6(b)). Therefore, the development of X-ray microscopy is very useful for imaging biological specimens with improved spatial resolution under wet and normal pressure conditions (Figure 6(a)) [88]. Besides, the X-ray energies of 10-100 keV cover the spectroscopic features of all elements and offer the opportunity to detect elements and probe chemical bonds of an object [89, 90].

### 6.2. The Optics in X-Ray Microscopy

In the late 1940s, the invention of grazing incidence mirror optics offers a great opportunity to develop X-ray microscopy. However, the technical issues of long exposure time and insufficient spatial resolution become a major challenge for the use of X-ray microscopy. In the 1970s, the development of high-quality zone plates for high-energy X-ray focusing opens the modern era of X-ray microscopy. The high-quality X-ray focusing optics are then extensively used to increase the spatial resolution of X-ray imaging. Nowadays, X-ray optics can be well designed by combination with thin-film deposition, electron beam lithography, and nanofabrication with capabilities to improve diffractive, reflective, and refractive X-rays [91]. The Fresnel zone plates consist of several concentric rings of transparent zones and alternating opaque, as shown in Figure 6(c) [92]. The X-rays passing through the transparent sections are diffracted and subsequently generate constructive interference, focusing on a small spot. Hence, the zone plates can be used both as condenser and objective for X-ray focusing. The zone plate-based microscopy is achievable for high-resolution imaging, which is largely determined by the zone plate's outer width (Δ*r*_*N*_). The smaller outer zone width can lead to a higher spatial resolution. The selection of the type of zone plate is determined by several factors including photon energy, required spatial resolution, and the number of zones. At present, a 12 nm spatial resolution has been successfully performed using the 12 nm zone plate [93].

The efficiency and resolution of hard-X-ray focusing are also achieved using a multilayer Laue lens with varied *d*-spacing, a multilayer coating-based 1D zone plate fabricated by magnetron sputtering [95]. As shown in Figure 6(d), the multilayer Laue lens are tiled to meet the Bragg condition for the outer, smallest layer spacing, providing efficiency larger than conventional Fresnel zone plates. Multilayer Laue lens of a 16 nm width was used to focus 20 keV photon energy of X-rays. Recently, a focal spot size smaller than 10 nm was achieved by fabricating multilayer Laue lenses with sufficiently high numerical aperture. Besides, for 2D focusing, the two multilayer Laue lenses with different focal lengths are required to be positioned orthogonal to each other.

Reflective optics are further developed to achieve the imaging resolution of several nanometers by exploiting Kirkpatrick-Baez systems (Figure 6(e)). Grazing incidence reflective mirrors are capable of focusing hard X-rays and enhancing X-ray reflection efficiency. Kirkpatrick-Baez mirrors are typically made from multilayers of dense metals or hard silicon carbide coated on silicon crystals with near atomic roughness. The efficiency of the Kirkpatrick-Baez mirror is constrained by the shape and surface roughness.

Since the refractive indices of all materials for X-rays are always slightly less than ones in vacuum and air, conventional optical refractive lenses are not available for X-ray focusing. In addition, an appropriate curvature radius and a double concave shape are essential for X-ray focusing lenses, as illustrated in Figure 6(f). In 1996, compound refractive lenses were designed using parabolic concave lenses [96]. To reduce X-ray absorption and increase compound refractive lenses' efficiency, compound refractive lenses are typically made of high-density, low-*Z* materials, such as lithium, boron, silicon, carbon, beryllium, or aluminum. Advantages in simple manufacture, low cost, small size, easy alignment, and tunable focal length make the compound refractive lenses great promise in hard X-ray focusing.

### 6.3. General X-Ray Microscopy Modes

Current state-of-art X-ray microscopy includes full-view transmission X-ray microscopy and scanning transmission X-ray microscopy [97, 98]. The full-view transmission X-ray microscopy is similar in principle to that of optical bright-field microscopy. X-rays travel through the focusing optics to irradiate the sample, and the transmitted X-rays are magnified by a zone plate to provide a magnified projection onto the detector [94]. As shown in Figure 6(g), the central stopper coupled with an order sorting aperture is applied to filtrate a certain portion of X-rays which is not in the first-order diffraction. The sample is placed near the spot of the first-order diffraction. The X-rays penetrating out from the samples are magnified using the microzone plates as an objective, projecting onto the X-ray detector. The imaging resolution of the full-view transmission X-ray microscopy depends on the outer zone width (Δ*r*_*N*_) of the zone plate, imaging geometry, and illumination coherence. Since the quick acquisition of a 2D projection image, a 3D image is reconstructed by many 2D projection images from different angles of the sample.

The scanning transmission X-ray microscopy is another technology suitable for imaging the local scale structure. Figure 6(h) shows the schematic setup of the scanning transmission X-ray microscopy. A coherent part of X-rays from a monochromator passes through the zone plate to produce a diffraction-limited focal spot, and then the transmitted X-rays are detected. As a result, scanning transmission X-ray microscopy image is reconstructed since the sample is scanned in 2D perpendicular to the optical axis. The imaging resolution is determined by several factors, including the quality of focusing lenses, the precision of instrumental setup, and the coherence of X-rays. The imaging resolution can be improved by a higher-order focusing zone plate, while the detection efficiency will be reduced. One key advantage of scanning transmission X-ray microscopy is its easy extension to multisignal, simultaneous detection in combination with X-ray scattering, diffraction, fluorescence, or electron emission yield [99].

## 7. Material Opportunity for X-Ray Imaging

The rapid development in materials science offers a great opportunity to revolutionize the future of X-ray imaging technology. Over the past decades, scintillator materials, which can convert high-energy radiation into UV-Vis photons, are critical to high-performance X-ray imaging. In the early stage, CaWO_4_ and ZnS powders were widely used for X-rays imaging. After the 1940s, scintillator crystals (e.g. NaI: Tl, CsI: Tl, and Bi_4_Ge_3_O_12_) were gradually used for fabricating high-performance X-ray detectors such as commercial flat-panel detectors. However, conventional scintillators are synthesized through a solid-state method at high temperatures, resulting in large crystals that are unsuitable for manufacturing large-area, flexible X-ray detectors.

**Figure 7:**
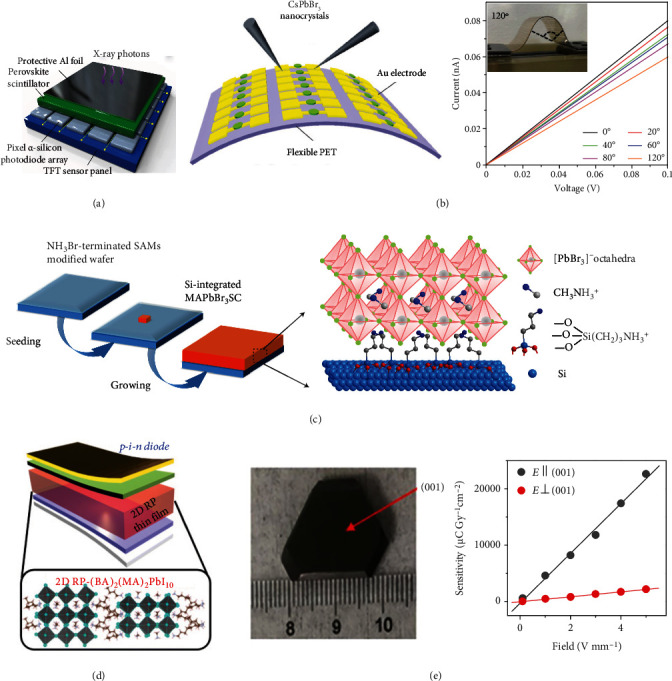
Perovskites-based X-ray detectors. (a) Schematic illustration of the perovskite nanocrystals-based flat-panel detector by coating a layer of CsPbBr_3_ nanoscintillators onto a commercial pixelated *α*-silicon thin-film-transistor (TFT) panel. (b) Schematic of a flexible perovskite X-ray detector (left) and *I-V* curves of the flexible device under X-ray irradiation (right). (c) Scheme illustrating the fabrication of Si-integrated MAPbBr_3_ single crystals. (d) Diagram of an X-ray detector based on 2D RP perovskite *p-i-n* thin-film. (e) Photography of a bulk (NH_4_)_3_Bi_2_I_9_ single crystal (left) and X-ray sensitivity measurement of (NH_4_)_3_Bi_2_I_9_ single-crystal device in the direction parallel and perpendicular to the (001) plane (right). (a) is reprinted with permission from ref. [104], copyright 2018 *Springer Nature Limited*. (b) is reprinted with permission from ref. [20], 2019 *WILEY-VCH Verlag GmbH & Co. KGaA, Weinheim*. (c) is reprinted with permission from ref. [60], copyright 2017 *Macmillan Publishers Limited*. (d) is reprinted with permission from ref. [107], copyright 2020 *American Association for the Advancement of Science*. (e) is reprinted with permission from ref. [110], copyright 2019 *Springer Nature Limited*.

Recently, solution-processed materials have been developed for advancing next-generation X-ray imaging technologies with low cost, high sensitivity, and flexibility. In particular, perovskites, featuring tunable bandgap, high photoluminescence quantum yields, narrow emission, and high charge-carrier mobility, have emerged as promising materials in photovoltaic devices, luminescence displays, and radiation detection [100–103]. The heavy atom-contained perovskites with efficient X-ray absorption show great potential in X-ray imaging applications. Lead-halide perovskite nanocrystals can generate multicolor radioluminescence upon X-ray irradiation [104]. The solution-processable and easily scalable CsPbBr_3_ nanocrystals are synthesized to fabricate large-area flat-panel X-ray detectors (Figure 7(a)). In addition, CsPbBr_3_ nanosheets synthesized at room temperature can be assembled into a uniform and dense thin film as an X-ray scintillating screen for high-resolution radiography [105]. Although indirect conversion-based X-ray detectors are most popular in practical applications, they generally suffer from a relatively low spatial resolution due to the optical crosstalk among neighboring pixels.

X-ray imaging detectors based on direct conversion present the advantages of a high signal-to-noise ratio and high resolution since the X-ray-generated charges can be directly collected by pixelated arrays [106]. Liu et al. fabricated a flexible X-ray detector using solution-processable perovskite nanocrystals by an inexpensive inkjet printing method (Figure 7(b)) [20]. Tsai et al. put forward an ultrasensitive X-ray detector by fabricating Ruddlesden-Popper (RP) layered perovskites in a fully depleted *p-i-n* architecture (Figure 7(d)) [107]. In 2015, Yakunin et al. reported that perovskite crystals of methylammonium lead iodide (MAPbI_3_) were developed to achieve indirect X-ray detection with strong X-ray absorption and high sensitivity [108]. Compared to the commercial direct conversion X-ray detectors using amorphous selenide as a photoconductor, perovskites which feature low-cost, defect-tolerance, solution-processibility, and tunable bandgap hold great promise for X-ray imaging, which is presented in Figure 7(c) [60, 109]. Despite their great progress, lead-halide perovskites suffer from the issues of poor long-term stability, and the toxicity of lead composition is harmful to the environment and human health. The diversity in substitution strategies offers the structural and functional flexibility to synthesize lead-free perovskites (Figure 7(e)) [110]. As such, 2D and zero-dimensional perovskites are further developed for achieving X-ray detection through tailoring ionic radius, chemical composition, and coordination environment based on the classical structure of ABX_3_ perovskites [111]. Recent studies have shown that many heavy atom-contained double perovskites have merits of efficient X-ray absorption, short decay time, and high stability, ideal for X-ray imaging [106, 112, 113].

Another focus of recent research is developing flexible X-ray detectors that are applicable to 3D X-ray imaging of irregularly shaped objects. Very recently, lanthanide-doped fluoride materials prepared by wet chemical methods were developed for high-resolution, flexible X-ray luminescence extension imaging. These materials prolonged radioluminescence and X-ray memory after the stoppage of the X-ray source, making it possible to fabricate flexible X-ray detectors [114]. After rational surface coating, the persistent luminescence intensity was enhanced by 6.5-fold, suggesting that the surface passivation can efficiently block the pathway of energy quenching by defects on the surface. The X-ray energy trapping capability and solution processibility allow fabricating the flexible X-ray detectors through embedding the nanoscintillators into the soft substrate, which is promising for portable X-ray devices, point-of-care radiography, and nondestructive testing in special conditions [115].

Apart from the inorganic scintillators, metal-free organic scintillators display great potential in large-area and flexible X-ray detectors, by taking advantage of flexibility, solution-processability, transparency, and ease to large-area fabrication. To date, the scientific community mainly focuses on developing lanthanide-doped materials, perovskites, and metal organic frames [116]. Considering that organic scintillators composed of carbon, hydrogen, oxygen, and nitrogen elements show a relatively low X-ray attenuation coefficient, the radioluminescence of organic scintillators can be brightened by introducing heavy atoms (such as chlorine, bromine, and iodine) to turn on the triplet excitons [117]. Overall, the emerging advanced materials present opportunities for promoting X-ray imaging technology with low-dose, high-resolution, and portability, and the performance of X-ray imaging can be improved in the terms of device physics, materials, and manufacturing methods.

## 8. Conclusion and Perspectives

X-ray imaging technology has been rapidly developed for various applications since 1895, offering new opportunities to scientific and industrial communities. Considering the fundamental and technical advances of X-ray detectors, we have summarized various X-ray working mechanisms that are crucial for specialized applications. The contrast-based X-ray imaging using a screen-film scintillation screen is a classical technique that greatly advances noninvasive medical imaging. The emergence of computed radiography has led to the technological evolution for digital X-ray imaging with more precise and instant information, while its separated readout mechanism suffers from technical limitations such as a high radiation dose and nondynamic imaging. Since the pioneering study in the 1990s, flat-panel X-ray detectors have been most prominent for achieving real-time digital radiography, which is popularly used in hospitals and industries in place of traditional computed radiography. In further development, CT integrating advanced helical scanning techniques and image reconstruction techniques is capable of providing comprehensive 3D structure information, which is a well-established cardiac, pectoral, and encephalopathic imaging modality with widespread acceptance and application.

Despite great efforts and tremendous achievements made in the past decades, the field of X-ray imaging is still in search of low-dose, high-resolution, large-area, flexible X-ray detectors. A low radiation dose used for X-ray imaging is an important technical consideration that people are always pursuing. One important aspect is to search advanced X-ray energy converting materials, which are critical for achieving efficient X-ray scintillating to increase the sensitivity of X-ray detectors. To date, the most efficient scintillators are limited to bulk CsI: Tl and GOS: Tb phosphors, while suffering from the drawbacks such as harmful scintillation decay, harsh synthesis process, and unsatisfied light yields. Another challenge that X-ray imaging faces are achieving a high spatial resolution for practical radiography due to the optical crosstalk on the transistor and the low sensitivity of the X-ray detectors. The combination of a high-efficiency X-ray converting layer and metasurface technology may be a promising strategy. In addition to the general considerations described above, one of the tremendous interesting directions is to develop large-area and flexible X-ray imaging detectors for potential applications in dental X-ray inspections, imaging of irregular objects, portable X-ray testing, and so on. The recent development outlined in this review is expected to stimulate future investigations for next-generation X-ray imaging technologies.
